# *Lactobacilli spp.:* real-time evaluation of biofilm growth

**DOI:** 10.1186/s12866-020-01753-3

**Published:** 2020-03-24

**Authors:** Stacy Martinez, Jonathan Gomez Garcia, Roy Williams, Moamen Elmassry, Andrew West, Abdul Hamood, Deborah Hurtado, Brent Gudenkauf, Gary Ventolini, Natalia Schlabritz-Loutsevitch

**Affiliations:** 1 Texas Tech University Health Sciences Center at the Permian Basin, 701 W. 5th Street, Odessa, TX 79763 USA; 2 University of Texas at the Permian Basin, Odessa, TX USA; 3 Department of Biological Sciences, Texas Tech University, Lubbock, TX USA; 4 Department of Microbiology and Immunology, Texas Tech University Health Sciences Center, Lubbock, TX USA; 5 ACEA Biosciences, Inc., San Diego, CA USA; 6 Department of Neurobiology and Pharmacology, Texas Tech University Health Sciences Center, Lubbock, TX USA

**Keywords:** Real-time detection, Biofilm, Lactobacilli, Micro-fermenter, xCELLigence, RNA-seq

## Abstract

**Background:**

Biofilm is a fundamental bacterial survival mode which proceeds through three main generalized phases: adhesion, maturation, and dispersion. *Lactobacilli* spp. (*LB*) are critical components of gut and reproductive health and are widely used probiotics. Evaluation of time-dependent mechanisms of biofilm formation is important for understanding of host-microbial interaction and development of therapeutic interventions. Time-dependent *LB* biofilm growth was studied in two systems: large biofilm output in continuous flow system (microfermenter (M), Institute Pasteur, France) and electrical impedance-based real time label-free cell analyzer (C) (xCELLigence, ACEA Bioscience Inc., San Diego, CA). *L. plantarum* biofilm growth in M system was video-recorded, followed by analyses using IMARIS software (Bitplane, Oxford Instrument Company, Concord, MA, USA). Additionally, whole genome expression and analyses of attached (A) and dispersed (D) biofilm phases at 24 and 48 h were performed.

**Results:**

The dynamic of biofilm growth of *L. plantarum* was similar in both systems except for D phases. Comparison of the transcriptome of A and D phases revealed, that 121 transcripts differ between two phases at 24 h. and 35 transcripts – at 48 h. of M growth. The main pathways, down-regulated in A compared to D phases after 24 h. were transcriptional regulation, purine nucleotide biosynthesis, and L-aspartate biosynthesis, and the upregulated pathways were fatty acid and phospholipid metabolism as well as ABC transporters and purine nucleotide biosynthesis. Four *LB* species differed in the duration and amplitude of attachment phases, while growth phases were similar.

**Conclusion:**

*LB* spp. biofilm growth and propagation area dynamic, time-dependent processes with species-specific and time specific characteristics. The dynamic of *LB* biofilm growth agrees with published pathophysiological data and points out that real time evaluation is an important tool in understanding growth of microbial communities.

## Background

Recent development of the concept of the microbiome has opened the question of co-existence of bacterial communities and mammalian cells [1] as well the presence of bacterial communities as an essential component for host function and survival [2, 3]. In the body, biofilm formation represents a major mode of bacterial colonization that can spread out over 300–400 m^2^ of the surface areas in humans [4, 5]. Growth of bacterial biofilms is a complex process involving three main phases: adhesion, biofilm maturation, and dispersion [1]. Adhesion starts with reversible and non-reversible attachment. The reversible process involves initial attachment and is driven by morphological, mechanical and electrical interactions with host cells [1, 6]. For the adhesion process, bacteria express multiple type of adhesions, e.g. cell appendages, or pili, which facilitate bacterial binding to host glycoproteins [7] and oligosaccharides [8]. The maturation phase is characterized by cellular division, production of the extracellular matrix, and dispersion [9]. The time-line of biofilm development by different bacteria is an important parameter [10]. Lactobacilli species (spp.) (*LB*) are abundant in human oral cavities [11], gut [12–14], vagina [15, 16] and milk [17]. *LB* have been shown to display therapeutic properties, e.g. in prevention of adhesion of *Trichomonas vaginalis* (by *L. gasseri*) [18], *Vibrio spp*., [19] and interfering with immune cells [20]. Thus, understanding any of the dynamics of biofilm growth by *LB* has high translational relevance. Despite the fact that *LB* biofilm has been described extensively [21–25], the temporal dynamic of biofilm growth of these species has yet to be evaluated. Here, the temporal dynamic of *LB* biofilm growth is described using two methods of label-free quantification: analyses of video-recording and electrical impedance-based technologies, as well as an additional RNA -sequencing was performed.

## Results

### Large scale biofilm growth

**Fig. 1:**
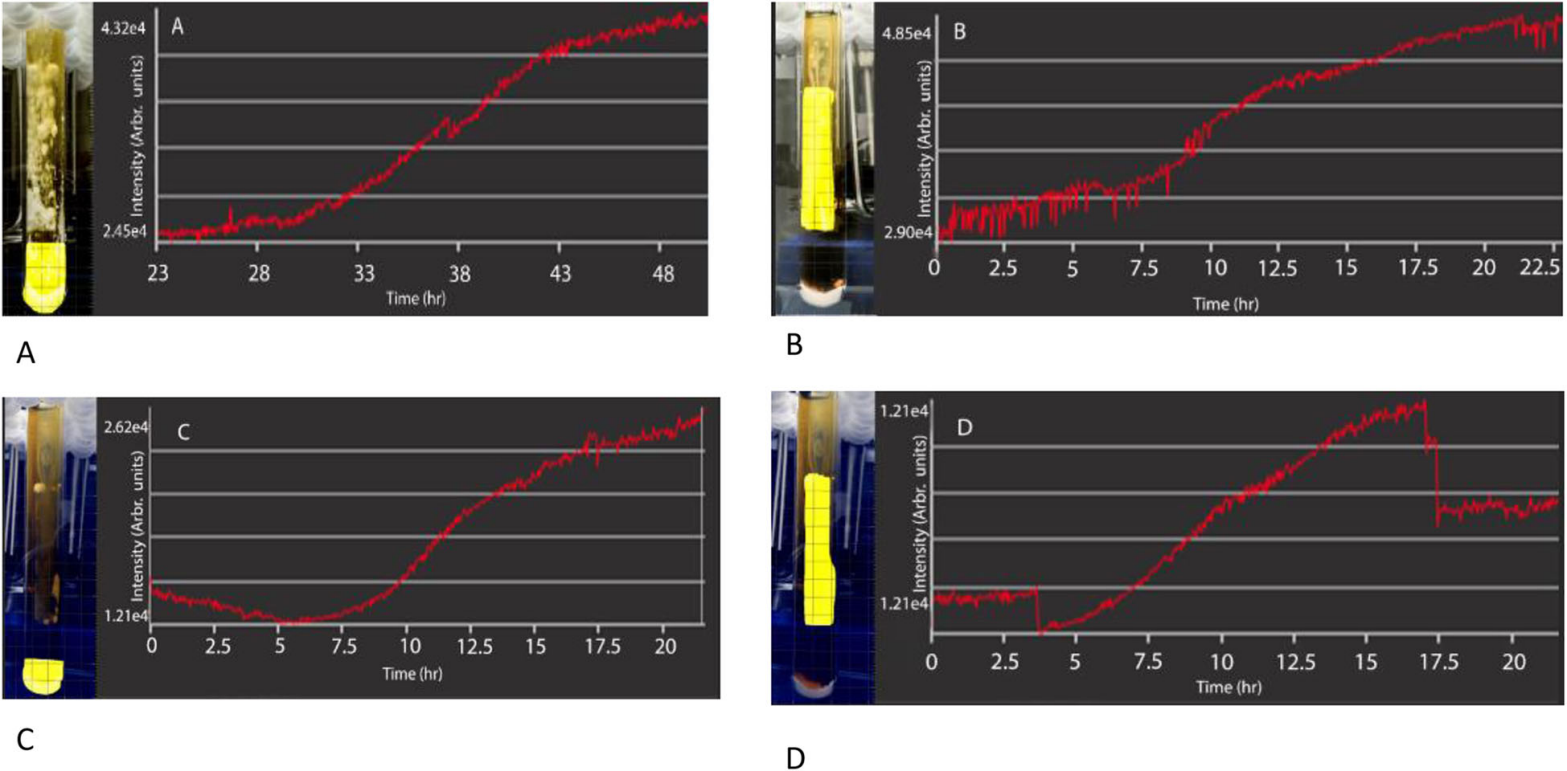
**a** Biofilm development for second 24-h timeframe. Intensity reaches a steady increase as the biofilm detaches from the spatula and moves to the bottom of the microfermenter. **b** Growth of the attached phase: Quantification of biofilm dynamics for the first 24 h of development. Initial intensity measurements are due to background impedance for selected region of interest (attached phase) **c** Growth of the detached phase: Growth initiates at about 6 h, and increases steadily due to spatula detachment, as well as growth from previous detachment. **d** Growth initiates at about 3.5 h, and the biofilm grows until it hits the detachment phase, at roughly 16 h. Once the detachment phase occurs there is a large drop off and then a constant plateau showing full biofilm maturation

**Fig. 2:**
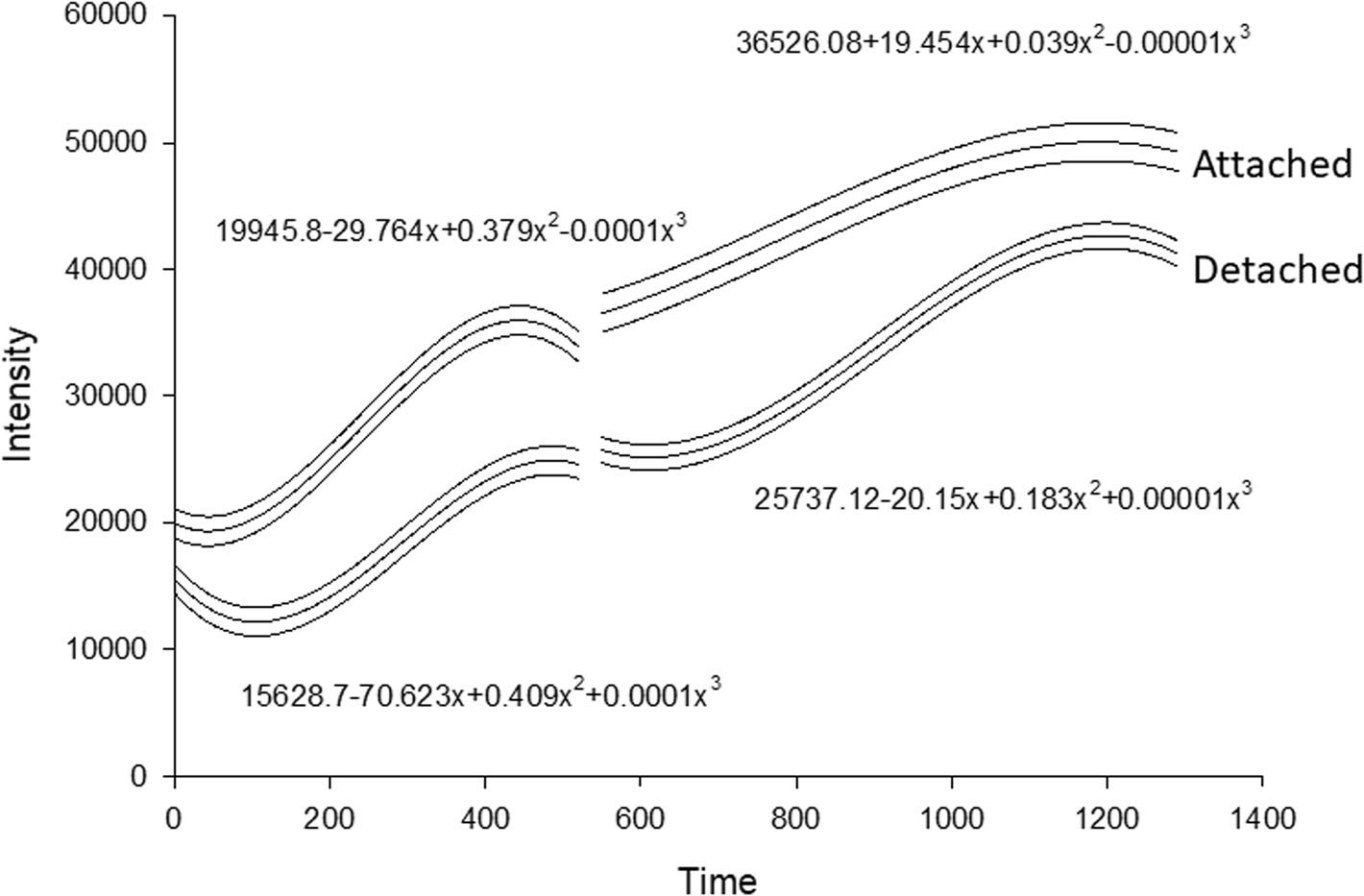
Function of intensity on time in the biofilm growth in microfermenter. The left panel shows the cubic equations, the lines represent mean and 95% confident intervals for each condition attached and detached. Notice there is not overlap between functions. Notice there is not overlap between functions. Intensity is measured in arbitrary units, and time (x – axis) correspond to the ranked samples for 48 h

The total final weight of the biofilm was 0.51 ± 0.09 g after 24 h. (*n* = 6) and 3.41 ± 0.26 g for 48 h. (*n* = 6). The weight of the attached phase was 0.46 ± 0.04 g (*n* = 5), and the weight of the detached phase was 3.13 ± 0.34 g (*n* = 5) at 48 h. Analyses of the video recording demonstrated growth of the attached phase, starting at 9 h. post- inoculation (Fig. 1a and b), followed by continuous growth, reaching a detachment/plateau at 42 h. The detached portion of the biofilm started to grow at 15 h. (Figs. 1c and 2d) and continued to grow, not reaching a plateau at 48 h. of recording. At approximately three-and-a-half hours, when formation of the biofilm had initiated, the software started to measure the biofilm and intensity, and the readout was corrected. At approximately 16 h. a large detachment was noted and reflected in fluctuations in intensity. New growth and development on the spatula were reflected on the rebound of the intensity at approximately 17. 5 h. (Supplementary material Video-recording S).

The best model for fit the intensity function was obtained with a cubic polynomial (R2 between 0.971 to 0.992) equation. The increase of intensity was clearly contrasted between the attached and detached conditions. The predicted model showed a slight difference between intercepts since basal measurement. However, after intercepts adjustment, the delayed signal intensity from detached condition remained during the whole experiment (Fig. 2).

### Total genome expression of sedimentary and detached phases of *L. plantarum* in large scale biofilm growth

**Fig. 3:**
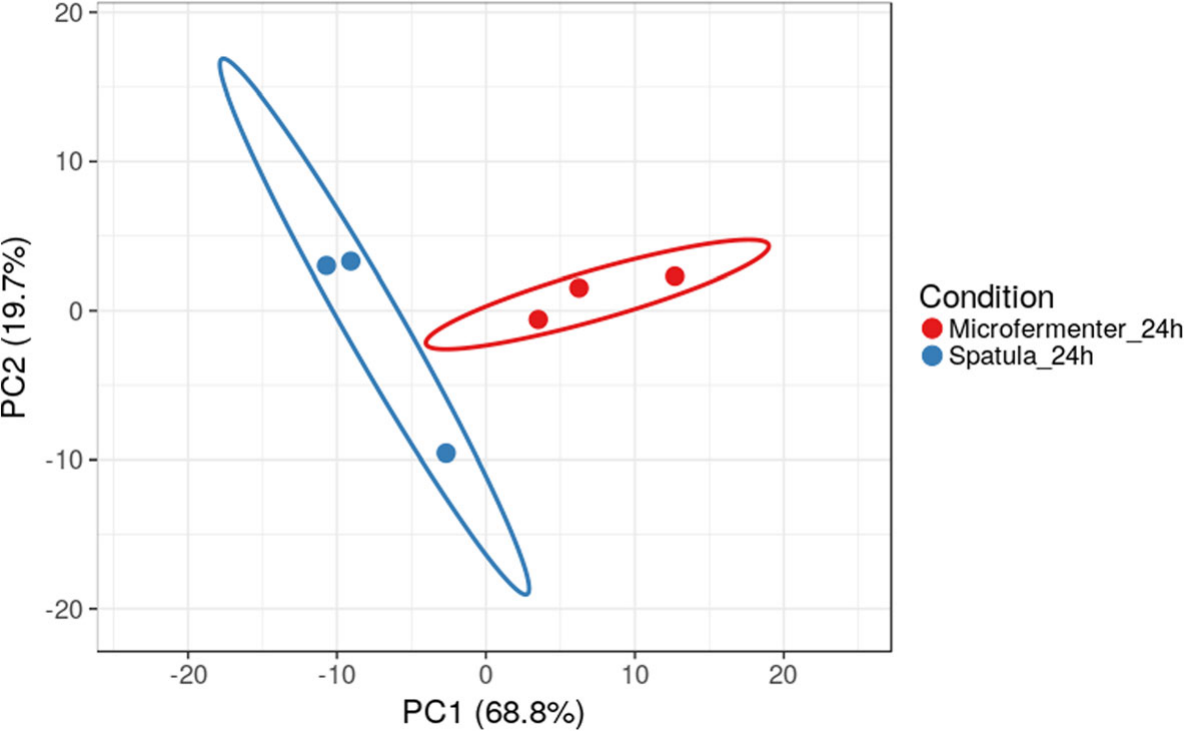
Principal component analyses of genes, expressed in biofilms, grown under different conditions (attached – spatula and detached (dispersed, microfermenter))

Comparison of attached and detached phases of biofilm growth revealed, that 121 transcripts differ between the two phases at 24 h. (Supplementary Table 2S) and 35 transcripts differ – at 48 h. of growth in micro-fermenter (Supplementary Table 3S). The main pathways, that were down-regulated in the attached phase after 24 h. in culture were transcriptional regulation, purine nucleotide biosynthesis, and L-aspartate biosynthesis, the upregulated pathways were fatty acid and phospholipid metabolism, ABC transporters (maltooligosaccharide, glutamine and oligopeptide transport systems) (Supplementary Fig. 3S), and purine nucleotide biosynthesis (Supplementary Fig. 4S). The Principal component analyses (PCA) demonstrated distinguishable differences between attached and detached phases after 24 h of biofilm growth (Fig. 3).

From 35 differently regulated transcripts at 48 h in culture, 33 molecules were down-regulated in A, compared to D phases, the main pathways affected were transcriptional regulation, fermentation and pyrimidine nucleotide biosynthesis. The comparison in gene expression in A phase of biofilm location revealed, that 29 genes were down-regulated and 2 genes were upregulated at 48 h.’ compared to 24 h.’ time-point. From the 29 down-regulated genes, 14 genes belonged to the purine synthesis pathway.

### Electrical impedance curves of different bacterial strains

**Fig. 4:**
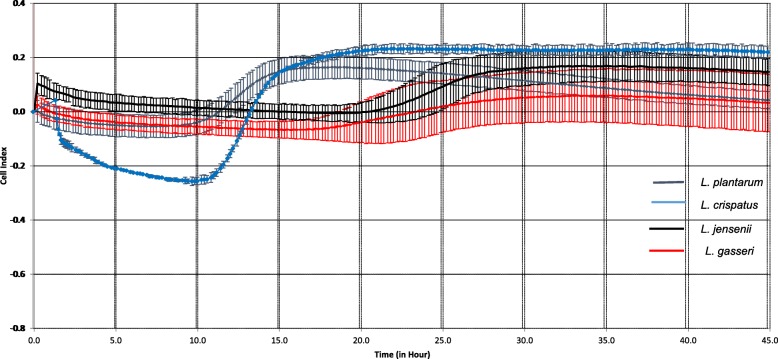
Biofilm growth curves of *L. plantarum*, *L. crispatus, L. gasseri* and *L. jensenii*, as changes of the Cell Index over time, detected with X-CELLIgence Real Time Cell Analyses Instrument (ACEA Bioscience Inc., San Diego, CA)

The growth of *LB* resulted in an initial decrease in CI, which was *LB* species-specific in amplitude and duration. This decrease was followed by an increase and subsequent growth plateau (Fig. 4, Table 1).

**Table 1:** Duration of attachment, growth and biofilm maturation and cell index of four *Lactobacilli species* (*L. plantarum, L. crispatus, L. jensenii and L.gasserii), detected, using X-Celligence methodology*

***Lactobacilli species***	Duration (h)		Cell Index		
	Attachment	Growth	Attachment	Growth slope	Maturation
*L. plantarum*	5.15	13.52	−0.3172	0.00745	0.717
*L. crispatus*	9.44	9.67	--0.26	0.0109	0.25
*L. jensenii*	19.52	13.95	−0.0261	0.00815	0.11
*L. gasserii*	12.22	12.27	−0.1224	0.0082	0.25

**Fig. 5:**
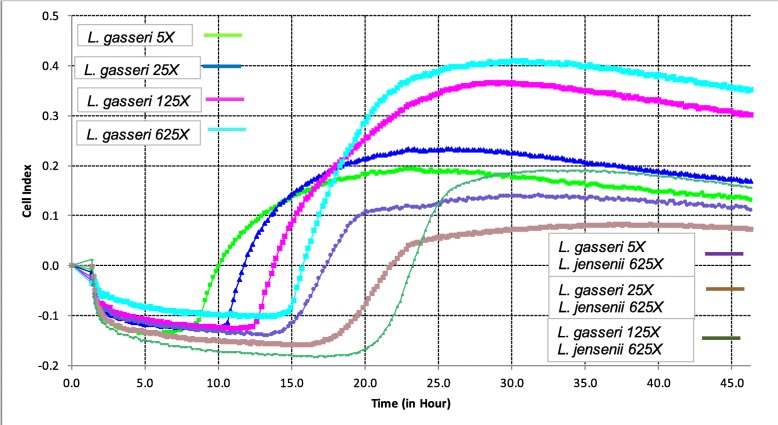
Biofilm growth curves of *Lactobacilli gasseri* in presence of *Lactobacilli jensenii* biofilm, X-CELLIgence Real Time Cell Analyses Instrument (ACEA Bioscience Inc., San Diego, CA)

The presence of diluted *L. jensenii* delayed the growth of *L. gasseri*. This inhibitory property of *L. jensenii* was demonstrated across a broad range of *L. gasseri* concentrations (Fig. 5).

## Discussion

*L. plantarum* is associated with oral and gut microbiomes and plays an important role in lipid metabolism [26], regulation of host growth [27], gut permeability [28], and antimicrobial defenses [29, 30]. *L. plantarum* has strong adhesive properties [23] and has been used as a probiotic for treatment of different conditions [31, 32] [33]. Time-curves of growth and maturation of *L. plantarum* were similar in both systems (microfermenter and XCELLigence); however, the detachment phase was not detectable in the impedance-based system in our study. This could be attributed to the differences between these two systems: the biomass, accumulated in microfermenter is much higher than what can be reached in the XCELLigencee. Additionally the microfermenter has a continuous flow, such that no medium exhaustion is taking place, as it might be the case in the different system. This allows formation of very mature biofilm, which could be thus detached. The micro-fermenter therefore might better reflect the real situation of biofilm formation in vivo with fluxes of liquid that are imposed in these natural environments with rarely some nutrients exhaustion rarely occurring. The detachment and dissemination took place at approximately 17 h. after initiation of the growth in the present study. Indeed, this time-frame is in line with the time of gastric emptying in humans [34]. The regulatory genes involved in the biofilm formation differ from those expressed in planktonic bacteria [5] and gene expression changes in bacterial biofilm over time [35]. Previous study of *L. plantarum* found several global regulators of gene expression, such as CtsR, HrcA, and CcpA, which upregulate the expression of various gene networks in response to stressors such as acidic or alkaline environments, high temperatures, and caloric restriction [36, 37]. These studies have indicated that, in response to stressors, *L. plantarum* changes its energy metabolism as well as its production of fatty acids and exopolysaccharides incorporated into biofilms [38, 39]. Our data agrees with these observations, demonstrating changes in oligosaccharide and fatty acid transports between attached (original place of biofilm formation) and detached phases after 24 h. in flow culture. Polysaccharides are critical for biofilm formation, comprising structures and supporting biofilm growth [5]. Additional factors, contributing to differences between attached and detached biofilm portions could be attributed to different fluid flow rates around the spatula and at the bottom of microfermenter [40] and differences in spatial morphology of biofilm [41]. Interestingly, previous work has shown that under severe stresses *L. plantarum* induces expression of error-prone DNA polymerases and may undergo “genetic shuffling” [42, 43]. Decrease in purine nucleotide synthesis in attached phase at 48 h. compared to 24 h. is in line with the time-line of biofilm formation, described in this study. Purine synthesis might be involved in regulation of *L. plant biofilm* growth in the similar manner as it has been described in other bacterial species. For example, in *Pseudomonas aeruginosa* mutation in purine synthesis gene slowed biofilm formation [44]. All of these mechanisms work synergistically to change gene expression to promote adaptation and survival, particularly to increase aggregation and adherence to surfaces, as well as metabolic change required for biofilm growth.

In general, the phases of the *LB* biofilm development detected in the ex vivo experiments should be taken in consideration for in vivo biofilm and probiotic application. Healthy vaginal microbiota includes different ratios of *L. crispatus, L. gasseri, L. jensenii* [45]. Using conventional methods, differences in biofilm formation by different *LB* strains were previously reported (e. g. crystal violet) [46]. These strain-specific *LB* biofilm dynamics might be related to the bacterial surface-specific properties encoded by each genome [47]. Remarkably, the duration of attachment phases differed between different *LB* while growth phases were quite similar. *L. jensenii* demonstrated the longest attachment phase and lower CI, these properties could be associated with the lower protective properties of this *LB,* which in turn might be associated with the dominance of *L. jensenii* in vaginal milieu of abnormal pregnancies (e.g. preterm birth) [48]. The inhibitory effect of *L. jensenii* on the growth of *L. gasseri* found in this study is in line with this clinical observation and the fact that the dominance of *L. gasseri* is associated with the decreased risk of preterm birth [49]. The biofilm interaction between *LB* species might represent the mechanisms of differential *LB* expression in Verheist criteria-determined vaginal milieu [50].

According to Martin et al., *L. jensenii* strains adhered strongly to a plastic substrate [51]. The shortest attachment period and highest absolute CI among vaginal *LB* was detected in *L. crispatus*. This result is in line with strong adhesive force reported for this species [46]. The differences in the phenotypic surface properties of three *LB* strains have been described by others [47], and agree with the findings noted in this study. Changes in the electrical impedance signal are bacterial species specific [52], thus this method could be used for development of the rapid diagnostic tool for bacterial detection of specific *LB* composition.

## Conclusions

The impedance-based technology could be used as a rapid screening tool for evaluation of competitive biofilm formation by different *LB* in co-cultures, and the microfermenter system could be used as a subsequent step for biofilm growth by specific strains and subsequent therapeutic applications, which are based on the general fact that *Lactobacilli* stimulate and support a dynamic and healthy gut and vaginal milieu by protecting against pathogens [36].

## Method

### I. Micro-fermenter

*L. plantarum* (subsp. *plantarum* (ATCC® 14917™)) was inoculated in a MRS (De Mann, Rogosa, and Sharpe) agar plate, which was subsequently incubated at 37 °C for 24 h. with 5% CO_2_. The culture was then scraped into 6 ml of an MRS broth. The Optical Density (OD) of the cell suspension was estimated using an EL 808 plate reader at 630 nm (Biotek, Winooske, Vermont, USA). A 1:10 OD dilution was made into a 50 ml culture that was then used to inoculate a spatula in the micro-fermenter. The spatula remained submerged in the culture broth for 30 mins and was then placed in the micro fermenter tube. The tube was, in turn, was in turn submerged in a 37 °C heating water bath in the continuous-flow culture system for either 24 (*n* = 6) or 48 h. (*n* = 6). A peristaltic pump (Multichannel cassette pump, Watson-Marlow Inc., Wilmington, MA, USA) was utilized at 10 rpm to push fresh MRS broth media through the system, which was pressurized with a mixture of 95% O_2_ and 5% CO_2_ (0.4 bar).

After 24 and 48 h. the biofilm was then collected and stored for further analysis at − 80 °C. The purity of the culture was confirmed by Polymerase Chain Reaction (PCR), using Fast Start Essential DNA Green Master Mix (Roche, USA) with specific primer sets (Supplementary Table 1S), using Roche Light Cycler® 96 (Applied Biosystems/Roche, USA).

#### Video recording

Real-time capture of the biofilm growth (*n* = 6: for full 48 h (*n* = 4) and for 24 h’ time-frames (*n* = 2)) was obtained using a Nikon D3200 (Nikon Inc. Melville, N. Y, U.S.A) digital camera. The photo-camera was placed externally in front of the micro-fermenter system. An external auto-timer shutter release (Shenzhen Pangshi technology, Ltd., Longhua Shenzhen, China) was set on a continuous cycle for image capture at a frequency of one frame every 2.5 min. Images taken made during these time-points were organized, edited, and exported using Adobe Photoshop Lightroom (Adobe Systems Incorporated, San Jose, CA). Each time-point was then put together with Adobe Premiere Pro (Adobe Systems Incorporated, San Jose, CA) to create a full video clip of the dynamic stages of the biofilm development. The video was then transferred to the IMARIS software for further quantification.

#### Quantification of the time-lapse video-recording of biofilm growth, using IMARIS system

Analyses of the biofilm growth from video recordings (*n* = 5, for 24 and 48, attached and detached phases) were performed using an algorithm developed by IMARIS Microscopy Image analyses software (Bitplane, Oxford Instrument Company, Concord, MA, USA). Regions of interest were selected for both the spatula (attached phase, A) and the bottom of the microfermenter (detached phase, D) for data collection and quantification. This also allowed the software to focus only on intensity changes within the given parameters and region. The initial intensity reading was performed at time-point 0 and defined as a ‘background noise.” Corrections were also made for the heating coil and bubbles attached to the glassware, which were picked up by IMARIS as biofilm intensity in the selected region of interest.

### Mathematical modelling

The intensity as a function of time demonstrated a sigmoidal shape. We calculated the beta value (slope) on the linear part of the curve. Four mathematical models were tested for goodness of fit of the function intensity on time. The evaluated models were liner, quadratic, cubic and exponent. These models were analyzed during the first 24 h and in a second model (a third was with intercept adjustment at basal) after 24 h of 48 h of growth. The SPSS v26 software was used for the analysis.

### RNA extraction and sequencing

Biofilm was collected separately from A and D phases after 24 (*n* = 3 each) and 48 h. (*n* = 3 each) of culture flash frozen in liquid nitrogen and stored at − 80 °C for future analyses. RNA was extracted, using TRIzol™ reagent [37] and Next Generation sequencing (NGS) was performed as previously described [38], utilizing the Illumina MiSeq platform (San Diego, CA). We compared whole genome expression of biofilms from the A and D phases at the bottom of the fermenter as validation of biofilm formation/maturation capacities as well as of biofilm growth by measuring both bacterial biomass from the spatula and from the bottom of the micro-fermenter in a time-dependent manner (24 and 48 h.).

### Analysis of the RNA-seq data

Rockhopper 2.03 software (https://cs.wellesley.edu/~btjaden/Rockhopper/) [39] was used to analyze RNA-seq data, implementing reference-based transcript alignment to *Lactobacillus plantarum subsp. plantarum* ATCC 14917 as a reference genome. Total transcriptomes were normalized by upper quartile normalization, then transcript abundance was quantified using reads assigned per kilobase of target per million mapped reads (RPKM). Differential gene expression was measured using local regression with an error term modelled with a negative binomial distribution. The Benjamini-Hochberg procedure [53] was used to correct for multiple testing and *q*-values were reported, which reflect adjusted *p*-values. Selection criteria for differential expression for genes included a fold change greater than 2 and a *q*-value less than 0.05. Kyoto Encyclopedia of Genes and Genome database (KEGG; https://www.genome.jp/kegg/) [42, 43] was used to map the identified genes to available and annotated pathways of *Lactobacillus plantarum* genome.

### II. xCELLigence assays

The xCELLigence (ACEA Bioscience Inc., San Diego, CA) system is based on impedance measurement of the cells, seeded on the 16 well plate with gold biosensors [54].

The strains *Lactobacillus_gasseri*_CIP102991T, *Lactobacillus_jensenii*_CIP69.17 T and *Lactobacillus_crispatus*_CIP103603 were obtained from the Institute Pasteur strains collection (CIP). Prior to the experiments, strains of *L. plantarum, L. jensenii, L. crispatus* and *L. gasseri* were inoculated and overnight bacterial culture was grown at 37 °C (5%CO_2_, 95% O_2_). The OD of the cell suspension was estimated using an EL 808 plate reader (Biotek, Winooske, Vermont, USA). The density of the seeding was estimated during preliminary experiments (Supplementary material, Fig. 2S). Each well of the xCELLigence E-Plate 16 PET (ref # 00300600890 from ACEA Biosciences, Inc., San Diego, CA, USA) was filled with 100 μl media and cells were seeded at the optimal density. Additionally, *L. jensenii* and *L. gasseri* were seeded in the same wells to evaluate competition between these *LB* subspecies during biofilm formation (one run, *n* = 2). Since each bacterial biofilm had a specific impedance curve, bacterial identification was performed based on the curve analyses. The impedance was registered as a Cell Index (CI), which was detected by the gold electrodes, placed on the bottom of the 16-well plate. The instrument measures the strength of attachment of the biofilm layer and the relative impedance is recorded in user-defined intervals continually in real time from the incubator.

The set up allowed simultaneous experiments in three 16 well plates. Each experiment was performed twice and in duplicate for each run. The principles of the assay are described elsewhere [54]. The growth slope (for attachment and proliferation phases) and Maximal CI (for mature biofilm) have been measured.

## Supplementary information

**Additional file 1 Table 1S.** Primers’ set used for the conformation of *L. plantarum* biofilm purity.

**Additional file 2 Table 2S.** Comparison of whole genome expression between attached and detached phases of biofilm growth of *L. plantarum* at 24 h.

**Additional file 3 Table 3S.** Comparison of whole genome expression between attached and detached phases of biofilm growth of *L. plantarum* at 48 h. and 35 transcripts differ – at 48 h. of growth in micro-fermenter (Supplementary Table 2).

**Additional file 4 Figure 1S.** Representation amplification curves (A) and melting picks (B) of PCR amplification products of *L. plantarum (*housekeeping gene and eubacteria specific primers) used for conformation of biofilm purity.

**Additional file 5 Figure 2S.** Electrical impedance signaling expressed as cell index, registered using xCELLigence assays of *L. plantarum* biofilm growth at the initial dilutions 5x, 25x, 125x and 625x of overnight bacterial culture. The most robust signal was obtained by seeding the lowest number of cells (i.e. the 625x dilution is the best, while the 5x dilution is the worst).

**Additional file 6 Figure 3S.** The upregulated ABC transporters pathway (maltooligosaccharide, glutamine and oligopeptide transport systems) in attached compared to the detached phases of biofilm growth after 24 h.

**Additional file 7 Figure 4S.** The upregulated purine nucleotide biosynthesis pathway in attached compared to the detached phases of biofilm growth after 24 h.

**Additional file 8 Video-recording S.** Video-recording of *L. plantarum* biofilm growth in microfermenter system during 48 h.

## Data Availability

All data generated or analyzed during this study are included in this published article [and its supplementary information files].

## Abbreviations

A: Attached phase, growth on the spatula in microfermenter system
CI: Cell index
D: Detached phase, growth on the bottom of the microfermenter
*LB*: Lactobacilli species
Spp: Species
