# Supplementary material for: Lactobacilli spp.: real-time evaluation of biofilm growth
Source: BMC Microbiol. 2020 Mar 24;20:64. doi: 10.1186/s12866-020-01753-3 (PMC7092459; doi:10.1186/s12866-020-01753-3)
Supplement: Supplementary file 1 — Additional file 1 Table 1S. Primers’ set used for the conformation of L. plantarum biofilm purity. [file 12866_2020_1753_MOESM1_ESM.docx]

**Table 1. Specific Primer sets, used for identification of purity growth of microfermenter**

| **L Plant F** | TTACATTTGAGTGAGTGGCGAACT |
| --- | --- |
| **L Plant R** | AGGTGTTATCCCCCGCTTCT |
| **recA F** | GGCAGAACAGATCAAGGAAGG |
| **recA R** | TATCCACTTCGGCACGCTTA |
| **UniF340 (Enterobacteriaceae)** | ACTCCTACGGGAGGCAGCAGT |
| **UniR514 (Enterobacteriaceae)** | ATTACCGCGGCTGCTGGC |
| **Uni515 F (E. coli)** | GTGCCAGCMGCCGCGGTAA |
| **Ent826 R (E. coli)** | GCCTCAAGGGCACAACCTCCAAG |
