# Supplementary figures and images for: Lactobacilli spp.: real-time evaluation of biofilm growth
Source: BMC Microbiol. 2020 Mar 24;20:64. doi: 10.1186/s12866-020-01753-3 (PMC7092459; doi:10.1186/s12866-020-01753-3)

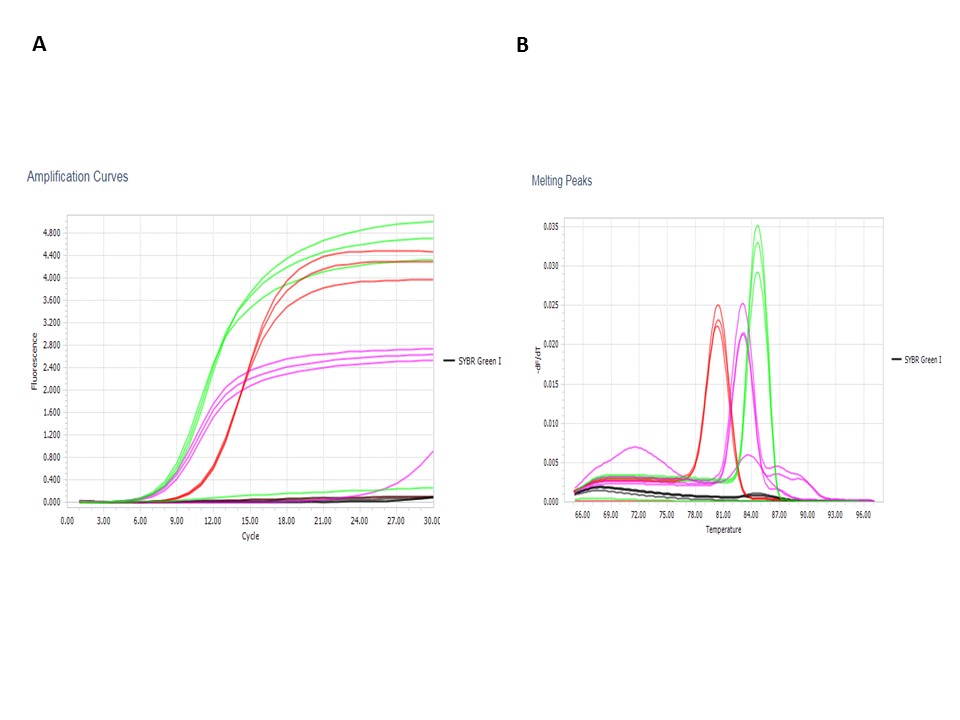

Supplement: Supplementary file 4 — Additional file 4 Figure 1S. Representation amplification curves (A) and melting picks (B) of PCR amplification products of L. plantarum (housekeeping gene and eubacteria specific primers) used for conformation of biofilm purity. [file 12866_2020_1753_MOESM4_ESM.jpg]

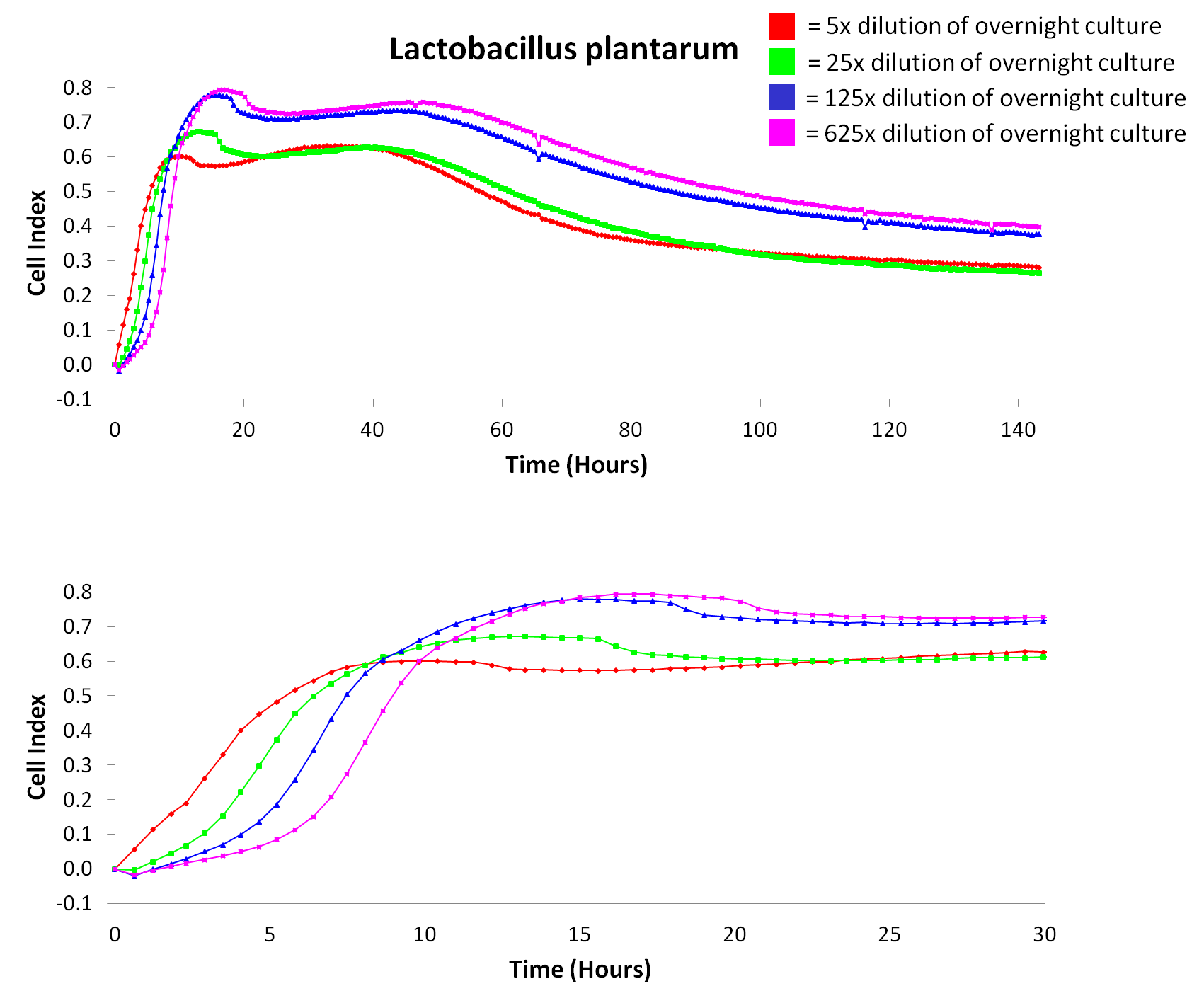

Supplement: Supplementary file 5 — Additional file 5 Figure 2S. Electrical impedance signaling expressed as cell index, registered using xCELLigence assays of L. plantarum biofilm growth at the initial dilutions 5x, 25x, 125x and 625x of overnight bacterial culture. The most robust signal was obtained by seeding the lowest number of cells (i.e. the 625x dilution is the best, while the 5x dilution is the worst). [file 12866_2020_1753_MOESM5_ESM.png]

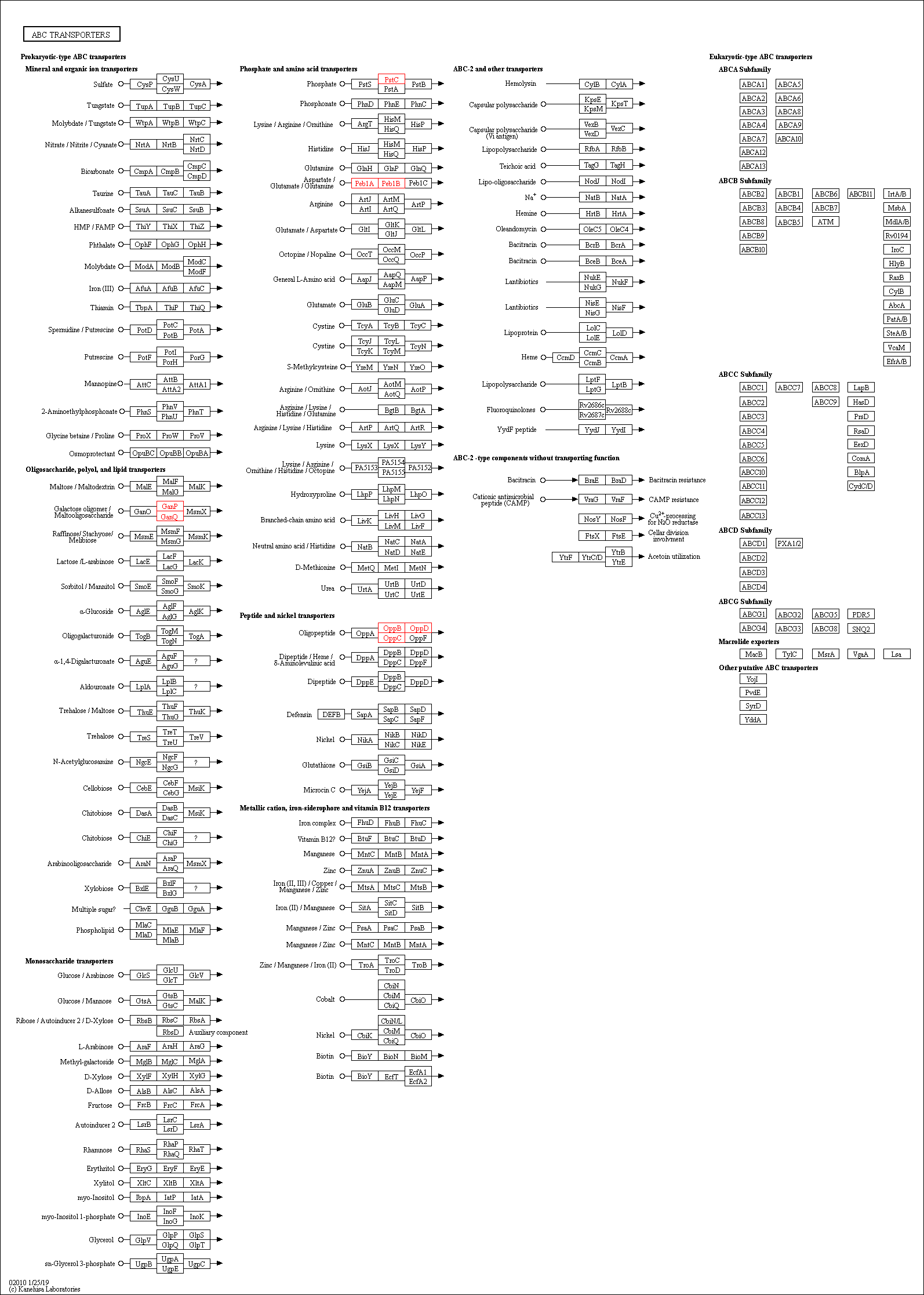

Supplement: Supplementary file 6 — Additional file 6 Figure 3S. The upregulated ABC transporters pathway (maltooligosaccharide, glutamine and oligopeptide transport systems) in attached compared to the detached phases of biofilm growth after 24 h. [file 12866_2020_1753_MOESM6_ESM.png]

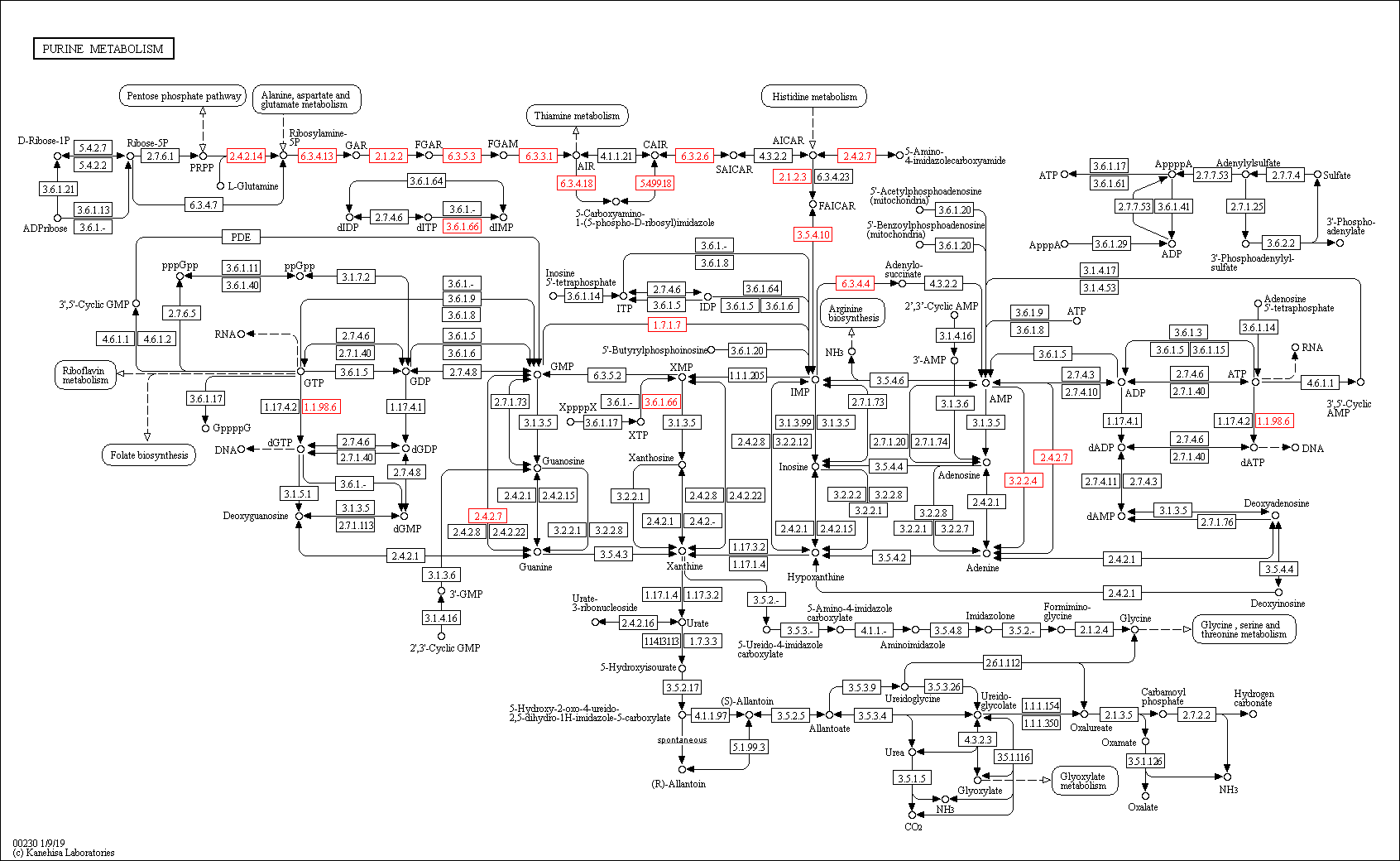

Supplement: Supplementary file 7 — Additional file 7 Figure 4S. The upregulated purine nucleotide biosynthesis pathway in attached compared to the detached phases of biofilm growth after 24 h. [file 12866_2020_1753_MOESM7_ESM.png]
